# Appointment of Dr. John Neill

**Published:** 1852-06

**Authors:** 


Dr. John Neill has been appointed Surgeon to the Pennsylvania Hospital. The hospital staff now consists of four surgeons and three physicians.

